# Prevalence and Associated Risk Factors of *Eimeria bovis* and *Eimeria zuernii* in Kacha Bira District, Central Ethiopia

**DOI:** 10.1155/2024/3145241

**Published:** 2024-03-21

**Authors:** Mesfin Mathewos, Habtamu Endale

**Affiliations:** ^1^School of Veterinary Medicine, Wachemo University, Hossana, Ethiopia; ^2^School of Veterinary Medicine, Wolaita Sodo University, Wolaita Sodo, Ethiopia

## Abstract

**Background:**

Eimeriosis, which is caused by several *Eimeria* species, is a protozoan disease affecting the cattle worldwide. The current investigation was aimed at ascertaining the prevalence of bovine eimeriosis, evaluation of the involved risk variables, and identification of the several *Eimeria* species that were prevalent in the Kacha Bira district of central Ethiopia.

**Methods:**

A cross-sectional coprological assessment of Bovine coccidiosis and its associated risk factors through a simple random sampling technique was conducted from January 2021 to December 2022.

**Results:**

The cumulative prevalence of coccidia was 17.83% (82/460) in the current investigation, with *Eimeria bovis*, *Eimeria zuernii*, and mixed infections having a respective prevalence of 7.83%, 3.25%, and 6.74%. From the anticipated risk factors, cattle age and months of the year have revealed a statistically significant (*p* < 0.05) association with the prevalence of eimeriosis in the cattle under investigation. However, there were no statistically significant (*p* > 0.05) relationships between the coccidia infection and the sex and breed of cattle and the season.

**Conclusion:**

During the study, a high prevalence rate of bovine eimeriosis was recorded. Thus, a further detailed study involving molecular techniques to identify prevailing *Eimeria* species is paramount to develop and put into effect evidence-based control strategies to tackle the prevalence and subsequent effect of eimeriosis.

## 1. Introduction

Particularly in Ethiopia, gastrointestinal parasites are thought to be the most prevalent diseases in cattle leading to animal loss and/or decreased productivity through mortality, morbidity, decreased growth rate, weight loss in calves which are still growing, late maturity of slaughter stock, decreased milk and meat production, and reduced animal working capacity [1–3].

Bovine eimeriosis is brought on by the widely distributed *Eimeria* species worldwide including *E. bovis*, *E. zuernii*, and *E. auburnensis* [4, 5] and is one of the most prevalent protozoan diseases in cattle managed in intensive farming systems [6, 7]. *E. zuernii* and *E. bovis*, on the other hand, are thought to be particularly dangerous and economically important in cattle, which result in financial loss through impaired performance, mortality, and the need for anticoccidial treatment. Reports show that the economic loss owing to bovine eimeriosis is USD 400 million across the world [8], USD 23.78 million from México, USD 62 million from the United States, and USD 3.8 million from Canada per annum [9].

The likelihood of cattle being infected by eimeriosis is determined by both animal-related factors such as the age of cattle, the dose of oocyst consumed, and the factors related to animal husbandry such as the management system and the presence of the oocyst in the area [10, 11]. It affects cattle of all ages [12, 13], but it is most common and serious in calves between the ages of three to six months [14, 15]. Relatively higher susceptibility of calf animals to eimeriosis than adults [16–18] is due to their underdeveloped immune systems which are not well-built or matured in order to respond sufficiently [19, 20]. The risk of infection and disease can also be increased by stressors such as weaning, dietary changes, harsh environments, inadequate nutrition, poor sanitation, and overcrowding [21]. The infection propagates in the herd and due to that the infected individuals continuously shed unsporulated oocysts along with their feces, resulting in contamination of the environment, which is the source for the subsequent infections. Once oocysts sporulate, they become infective and are protected from the environment by double cyst walls [22, 23]. Oocysts sporulate within 2–4 days in a temperature of around 27°C, with 10% of the normal amount of oxygen and 16% humidity but the time taken may vary according to temperature, moisture, and season [24–27]. These sporulated oocysts get in the cattle body as they ingest contaminated feed and water as well as when they groom themselves or others with oocysts on their hair coat [28].

Bovine coccidiosis is typically identified as a herd health issue rather than an issue of individual animals [29]. It is associated with widespread ailment in animals leading to significant economic losses through the cost of treatment, production reduction, low weight gain, and related means [30]. Compared to clinical form, subclinical eimeriosis which accounts for over 95% of all losses associated with eimeriosis is more important as it affects the animal without overt clinical signs hindering earlier disease identification and management and facilitating disease spread as the animal continuously sheds oocysts [31]. Reduced appetite, tiredness, weight loss, poor feed conversion, unthriftiness, diarrhea, anemia, dysentery, and anemia are all clinical signs of clinical bovine eimeriosis [16, 19].

Studies conducted in different parts of Ethiopia demonstrated that eimeriosis is highly prevalent with frequency ranging from 19.01% to 72.4% [15, 32]. It is important to keep in mind that Ethiopia is endowed with a huge population of livestock including 59.5 million cattle which put the country first in Africa and sixth in the world [33] but the productivity is retarded by different factors such as an infectious disease like coccidiosis [15]. There are numerous reports on bovine coccidiosis from different parts of Ethiopia [34], from Dire Dawa, eastern Ethiopia [15], from Bahir Dar, North West Ethiopia [35], from Asela town, Southeast Ethiopia [36], from Mekelle, northern Ethiopia [36], from Jimma town, Ethiopia [37], from Holeta, West Shewa Zone, Oromia [38], from Kombolcha district of South Wollo [39], and others. However, the status of bovine eimeriosis in this study area was not documented on the scientific web, and thus the current investigation intended to determine the prevalence of bovine eimeriosis, assess the contributing risk factors, and identify the prevailing *Eimeria* species in the Kacha Bira district of central Ethiopia.

## 2. Materials and Methods

### 2.1. Study Area

The current investigation was conducted in the Kacha Bira district, Kembata Tembaro Zone, central Ethiopia. With Durame as its capital, the Kembata Tembaro zone is one of the central Ethiopian zones. The specific study site, the Kacha Bira district is situated at a latitude of 7°10′–7°34′N, and the longitude of 37°58′–37°86′E 37°58′ to 37°86′E is where the district's altitude falls. The district's elevation ranges from 1650 to 2450 meters above the sea level. The district, with the exception of a few steep places, has topographically suitable terrain for agriculture. The major rainy season is from June to September, and there are variations in the yearly rainfall between 900 and 1500 mm. The average annual temperature is between 14 and 26°C [40]. There are six urban and twenty rural kebeles (peasant associations) in the woreda (district). The district has 133,303 people living in it as of 2010. There are 18,605 households in total, of which 15,238 are male and 3,367 of which are female. A total of 36,790 hectares of land are available, of which 21,875 hectares are suitable for agriculture. The region frequently uses semi-intensive management approaches for small-scale dairy farming. Local breeds were also grown in addition to exotic livestock, primarily Holstein Friesian [33, 40].

### 2.2. Study Animals

A total of 460 male and female cattle of various breeds and age groups randomly selected from various peasant associations in the districts were included in the investigation. The study cattle were divided into three groups: calves which are <1 year old, calves which are 1 to 3 years old, and adults and olds which are >3 years old according to Kemal and Terefe [41]. All cattle subjected to the investigation were kept under an extensive managed system mainly based on a free communal grazing system. While sampling, only a single animal was sampled from each herd selected randomly.

### 2.3. Study Design

A cross-sectional study was conducted from January 2021 to December 2022 to assess the associated risk factors, determine the prevalence of bovine eimeriosis, and identify the existing *Eimeria* species in Kacha Bira district of central Ethiopia.

### 2.4. Sampling Method and Sample Size Determination

The study animals were selected from different peasant associations (kebeles) through a simple random sampling technique, and the total number of sampled cattle was determined using the Thrusfield [42] formula as

(1) $$\begin{array}{c} N=\frac{\left[{\left(1.96\right)}^{2}*\text{Pexp}\left(1-\text{Pexp}\right)\right]}{d^{2}}, \end{array}$$

where *N* is the required sample size, Pexp is the expected prevalence (50%), and *d* is the desired absolute precision (0.05). Execution through the abovementioned formula gives the sample size of 384 cattle, but the number of the cattle sampled was raised to 460 in search of increasing precision of data used in the current study.

### 2.5. Study Method

#### 2.5.1. Coprological Examination

Thirty grams of faecal samples from each animal were collected directly by reaching the rectum or sometimes from freshly passed feces using a sterile disposable plastic glove (UltraPoly (MG950), Medgluv, USA). The samples were kept in a clean plastic container (BS EN 14254, UK) and labeled appropriately with the sampling date, identification number, age, sex, breed, owners' name, address, and season of sampling. Then, the sample was transported in a cool icebox (CLR-5, Infitek, co. ltd, China) to the Wolaita Sodo University Veterinary Parasitology Laboratory on the day of collection and preserved at refrigeration temperature of +4°C until processing within 48 hours of arrival. The presence of oocysts in faecal samples was examined with a flotation method using a saturated sodium chloride solution [43].

**Figure 1:**
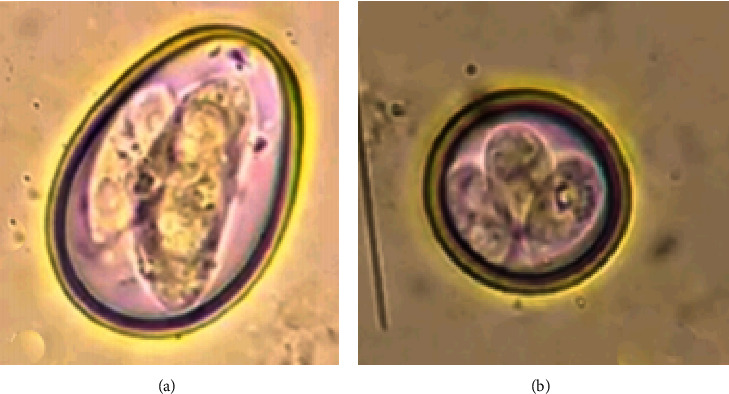
Microscopic figures of oocysts of *E. bovis* (a) and *E. zuernii* (b).

During the coprological examination, three grams of feces from each sample were mixed with 42 ml of flotation fluid. This mixture was thoroughly crushed using a pestle and mortar. Then, the suspension was poured through a tea strainer to remove any large particles. The filtered suspension was then transferred into a test tube and placed in a rack. The suspension was left undisturbed, allowing a convex meniscus to form at the top of the test tube. A cover slip was carefully placed on top of the tube and left to stand for 15 minutes. After that, the coverslip was removed, ensuring that any fluid adhered to it is retained. The cover slip was promptly placed on a microscopic slide and examined under 40x magnification to identify *Eimeria* oocysts [44]. The *Eimeria* species were identified based on the morphology of oocysts and sporocysts (shape, color, form index, micropyle and its cap, presence or absence of residual, and polar granule) and time of sporulation [43–45], with the morphological characterization of a minimum of 10 oocysts for each species [6]. Both species of *Eimeria* were identified by using the following morphological keys unique for each species. *Eimeria bovis* oocysts were relatively larger than that of *E. zuernii*, measuring approximately 30 to 50 *μ* in length and were ovoid-shaped with flattened ends, and their wall was smooth, thin, and colorless, and under a microscope, the oocysts appeared transparent. Internally, *E. bovis* sporocysts were relatively large, measuring approximately 23 to 28 micrometers in length, ovoid-shaped; similar to the oocysts, each oocyst contained four sporocysts, each with a single sporozoite, and sporozoites were elongated and slightly curved. In contrast, externally *E. zuernii* oocysts were relatively smaller compared to *E. bovis*, measuring on an average 23 to 30 micrometers in length, and oocysts were ovoid-shaped but tended to appear more rounded than *E. bovis*. Internally, sporocysts were smaller than *E. bovis*, measuring approximately from 15 to 17 micrometers in length, and elongated and slightly curved (Figure 1) [46–49].

### 2.6. Data Management and Statistical Analysis

The data collected on *Eimeria* species of cattle and its associated risk factors were entered into the Microsoft Excel worksheet 2016 and analyzed using STATA version 14. The prevalence of the *Eimeria* was explained by descriptive statistics such as percentages, and the associations between explanatory variables (risk factors) and status variables (outcome variables) were performed by the chi-square test. The association of individual risk factors with an outcome variable was screened by univariate logistic regression. A statistically significant association was considered at a *p* value of less than 0.05.

## 3. Results

Under microscopic examination of the faecal sample, the oocyst of *Eimeria bovis* which was an ovoid measuring approximately 30–50 *μ* in length with flattened ends, containing ovoid sporocysts of around 23–28 micrometers in length, where each oocyst contains four sporocysts, each with a single sporozoite, was observed (Figure 1(a)). *E. zuernii* oocysts which were relatively smaller than *E. bovis*, an average of 23–30 micrometers in length, rounded than *E. bovis*, and containing sporocysts of approximately 15–17 micrometers in length, were observed (Figure 1(b)).

### 3.1. Overall Prevalence of Eimeriosis in Cattle and Its Associated Risk Factors

The current investigation revealed that the overall prevalence of eimeriosis in cattle of the study site was 17.83% (82/460), consisting of *E. bovis* (7.83%), *E. zuernii* (3.25%), and mixed infection (6.74%), respectively (Table 1).

Based on the age group, eimeriosis in calves was recorded as the highest among the others, calves (1–3 yrs) (31.11%), followed by adults and old (20.49%) and calves (<1 yr) (10.53%) of the cattle. There was a statistically significant association between *Eimeria* infection and the age of the cattle (*p*=0.003). Eimeriosis was slightly higher in female cattle (18.33%) than in males (16.78%) (Table 2).

Regarding to the cattle breed, eimeriosis was more prevalent in Zebu breed (18.41%) than in those of cross-breed (Holstein Friesian × indigenous Zebu) cattle (9.68%). With regard to the season of the year when the study was undertaken, a higher infection rate of eimeriosis was recorded in the dry season (19.5%) than in the wet season (16.54%). Nevertheless, the difference between eimeriosis and sex, breed, or season was not statistically significant (*p* > 0.05) (Table 2). The odds of calves contracting eimeriosis were 1.8 times higher (CI: 0.59–5.46) than those of adults (CI: 0.13–0.94) while keeping old cattle constant. However, the odds of indigenous breeds of cattle suffering from eimeriosis were 8.97 times higher (CI: 0.35–227.40), while keeping crossbreed cattle constant. The odds of eimeriosis during the wet season were 0.69 times higher (CI: 0.12–4.07), while infection during the dry season was kept constant (Table 2).

### 3.2. Frequency of *Eimeria* Infection in Relation to the Months of the Year

Regarding the prevalence of *Eimeria* infection in cattle in the months of the year during which the investigation was conducted, the highest infection rate was observed in February (30%), followed by November (26%), December (20%), July (18.2%), October (18%), and September (14%), whereas it was lowest in August (7.27%). The difference among the months of the year was statistically significant (*x*^2^ = 16.07, *p*=0.041) (Table 3).

## 4. Discussion

Bovine coccidiosis is a well-known economically significant serious gastrointestinal parasite that damages the intestines of infected cattle [29, 50]. In the present investigation, 17.83% overall prevalence of eimeriosis was documented which was somewhat comparable with the previous reports of authors mentioned in references [34, 51–53] and [54], who reported 18.5% in Sekela district, 21.1% in and around Gondar area, 22.7% in Dire Dawa, 24.4% in Haramaya, and 22.9% Wolaita Sodo town, respectively. Conversely, it was lower than the previous reports of [55–59] and [60] who reported an eimeriosis prevalence of 39.7% in Veracruz, México; 32.17% in Punjab, India; 57.2% in Ravi River region, Lahore, Pakistan; 62.5% in Asella Oromia state, Ethiopia; 31.9% in Kombolcha Oromia state, Ethiopia; and 68.1% from Debre Zeit and Addis Ababa, respectively. This disparity in the prevalence of bovine eimeriosis in different regions is likely due to differences in agroecology, study season, and husbandry practices [15, 51].

In the present study, two pathogenic species of *Eimeria such as E. bovis* and *E. zuernii* were identified with their respective prevalence rate of 7.83% and 3.25% and with coinfection infection in both species (6.74%). This finding corroborates the previous reports of [5, 17, 58] and [61], who documented a high prevalence of *E. bovis* and *E. zuernii* in their study. In addition, other scholars [62, 63] also reported that *E. zuernii* and *E. bovis* were the most dominant species of *Eimeria* in cattle, but the infection owing to them was not clinical. This high prevalence of subclinical infections in infected cattle poses a detrimental impact on animal productivity culminating in financial losses attributed to poor feed conversion rate, retarded weight gain, body condition loss, unthriftiness, and increased vulnerability to other diseases [43, 51]. Furthermore, environmental contamination by incessant oocysts shed by subclinically infected cattle poses severe eimeriosis risk in highly vulnerable new cattle in these areas [64]. On the contrary, another authors [5, 12, 65] documented that *E. zuernii* and *E. bovis* were the most pathogenic and mainly associated with clinical coccidiosis in cattle. On the other hand, the authors in reference [59] reported that *Eimeria canadensis* was the most prevalent species of coccidia in Veracruz, México.

Based on the age of the cattle, the prevalence of eimeriosis was highest in calves (31.1%) followed by adults (20.4%) and old (10.53), age groups. This observation was consistent with the previous reports of [16, 51]. The odds of calves contracting eimeriosis and thus shedding Eimeria oocysts were 1.8 times (OR = 1.8; CI = 0.59–5.46) higher than those of calves cattle (OR = 0.35; CI = 0.13–0.94) while adult and old held constant. This was unswerving with the findings of [6, 17, 66]. The difference in the prevalence of eimeriosis among the different age groups of the cattle was statistically significant (*p* < 0.05); thus, age was an influential factor for the prevalence of bovine eimeriosis as reported by the authors in [15, 51, 58]. These may be accredited probably to the immaturity of the immune system of the calves compared to calves and adult animals [67, 68]. In addition, the authors in [12, 69] also pointed out that a higher incidence of eimeriosis in calves was associated with their poor immune system, which might lead them to higher susceptibility to *Eimeria* infection. In contrast, older cattle might produce antibodies during earlier challenges by the parasite making them more resistant to subsequent infections.

Concerning the seasons of the year, a higher prevalence of *Eimeria* infection was documented during the dry season (19.5%) than in the wet season (16.54%) in the current investigation. However, the difference was not statistically significant (*p* > 0.05) for the occurrence of *Eimeria* infection. This is most likely owing to the high parasite oocyst shading habit of cows during wet seasons, which is favorable for the oocyst survival and sporulation results in higher infection in subsequent dry seasons [13]. In relation to the sex of the cattle under investigation, the infection rate was almost related in both sexes, with 16.78% in males and 18.41% in females. Regarding the breed of cattle, a higher (18.41%) infection rate was recorded in local breeds than in cross breeds (9.68%). However, the difference was statistically insignificant (*p* > 0.05). In contrast, another authors reported that there was a statistically significant difference between both sexes, statistically significant higher prevalence in females [12] and statistically significant higher prevalence in males [15].

In relation to the breed of the cattle under study, a higher prevalence (18.41%) of coccidiosis was recorded in local breeds than in crossbreeds (9.68%), however the difference was statistically insignificant (*p* > 0.05). This lower prevalence rate in cross breeds may be attributed to the owner's husbandry habit in which people in the region study was conducted commonly manage cross breeds cows in a relatively hygienic and good husbandry. Furthermore, authors in [70, 71] stated that breed has a significant effect on the prevalence of coccidiosis. In relation to the months of the year during which the investigation was conducted, the highest infection rate of *Eimeria* was observed in February (30%), whereas it was lowest in August (7.27%). The prevalence of the disease has revealed statistically significant (*p* < 0.05) differences among the months of the year. As discussed earlier in this manuscript, a higher rain is favorable for the survival of *Eimeria* oocyst as the oocyst does not survive in dry climates [72–74].

## 5. Conclusion

The present study shows that the prevalence of eimeriosis was recorded high causing health problems for cattle in the Kacha Bira district. Among the probable risk factors, the age of cattle has shown a statistically significant difference with *Eimeria* infection. *Eimeria zuernii* and *Eimeria bovis* were the two species identified in the current study. Thus, a further detailed study involving molecular techniques to identify prevailing *Eimeria* species is paramount to develop and put into effect evidence-based control strategies to tackle the prevalence and subsequent effect of eimeriosis.

## Figures and Tables

**Table 1:** Overall prevalence of bovine eimeriosis.

Species of *Eimeria*	No. of cattle examined	No. of positive cattle	Frequency (%)
*E. zuernii*	460	36	3.25
*E. bovis*	460	15	7.83
Mixed	460	31	6.74
Total		82	17.83

**Table 2:** Univariate logistic regression of eimeriosis in cattle with associated risk factors.

Risk factors	No. of cattle examined	No. of positive cattle	Frequency (%)	OR	95% CI	*p* value
*Age*						0.003
Calves (<1 year)	45	14	31.1	1.8	0.59–5.46	
Calves (1–3 years)	171	18	10.53	0.35	0.13–0.94	
Adults and olds (>3 years)	244	50	20.49	1	1	
*Sex*						0.629
Male	149	25	16.78	0.82	0.35–1.95	
Female	311	57	18.33	1	1	
*Breed*						0.220
Local	429	79	18.41	8.97	0.35–227.40	
Crossbreed	31	3	9.68	1	1	
*Season*						0.411
Dry	200	39	19.5	1	1	
Wet	260	43	16.54	0.69	0.12–4.07	
Overall prevalence			17.83			

OR, odds ratio; CI, confidence interval.

**Table 3:** Prevalence of eimeriosis in cattle in months of the year.

Months	No. of cattle examined	No. of cattle infected	Prevalence (%)	Chi-square (*X*^2^)	*p* value
January	50	9	18	16.07	0.041
February	50	15	30		
March	50	5	10		
July	55	10	18.2		
August	55	4	7.27		
September	50	7	14		
October	50	9	18		
November	50	13	26		
December	50	10	20		
Total	460	82	17.82		

## Data Availability

The datasets used and analyzed during the current study are available from the corresponding author upon reasonable request.
